# Modeling the Effect of Alternative Cementitious Binders in Ultra-High-Performance Concrete

**DOI:** 10.3390/ma14237333

**Published:** 2021-11-30

**Authors:** Solmoi Park, Namkon Lee, Gi-Hong An, Kyeong-Taek Koh, Gum-Sung Ryu

**Affiliations:** 1 Department of Civil Engineering, Pukyong National University, 45 Yongso-ro, Nam-gu, Busan 48513, Korea; solmoi.park@pknu.ac.kr; 2 Department of Structural Engineering Research, Korea Institute of Civil Engineering and Building Technology, 283 Goyangdae-ro, Ilsanseo-gu, Goyang-si 10223, Korea; nklee@kict.re.kr (N.L.); agh0530@kict.re.kr (G.-H.A.); 3 Korean Peninsula Infrastructure Special Committee, Korea Institute of Civil Engineering and Building Technology, 283 Goyangdae-ro, Ilsanseo-gu, Goyang-si 10223, Korea; ktgo@kict.re.kr

**Keywords:** UHPC, thermodynamic modeling, phase assemblage, alternative cementitious binders, supplementary cementitious materials

## Abstract

The use of alternative cementitious binders is necessary for producing sustainable concrete. Herein, we study the effect of using alternative cementitious binders in ultra-high-performance concrete (UPHC) by calculating the phase assemblages of UHPC in which Portland cement is replaced with calcium aluminate cement, calcium sulfoaluminate cement, metakaolin or blast furnace slag. The calculation result shows that replacing Portland cement with calcium aluminate cement or calcium sulfoaluminate cement reduces the volume of C-S-H but increases the overall solid volume due to the formation of other phases, such as strätlingite or ettringite. The modeling result predicts that using calcium aluminate cement or calcium sulfoaluminate cement may require more water than it would for plain UHPC, while a similar or lower amount of water is needed for chemical reactions when using blast furnace slag or metakaolin.

## 1. Introduction

Ultra-high-performance concrete (UHPC) is considered one of the most promising construction materials in terms of performance. It is typically produced at low water-to-binder (w/b) ratios (0.15–0.25) and exhibits outstanding mechanical properties (compressive and tensile strengths exceeding 120 and 5 MPa after 28 days of curing) [1,2]. Due to the use of low w/b ratios, UHPC generally possesses extremely low porosity and excellent resistance to various chemical degradations. UHPC has been a topic of numerous studies which focused on various aspects of UHPC, such as its fresh and hardened state properties [3,4,5,6,7], effects of mix designs [8,9,10,11] and its performance at a structural level [12,13,14,15]. Due to the fact that the mixture proportioning and production methods of UHPC became mostly standardized in many countries, Portland cement (PC) is used as a cementitious binder along with silica fume in most cases. This is particularly important in recent years, where the high CO_2_ footprint of PC has been a global concern and the demand for more sustainable cementitious binders is dramatically increasing.

Some attempts have been made to investigate the effect of using alternative cements or supplementary cementitious materials in the replacement of PC in UHPC. Song et al. [16] studied the effect of calcium sulfoaluminate cement (CSA) addition on the microstructure of UHPC, and showed that CSA tends to reduce the autogenous shrinkage of UHPC, concluding that a 5–15% addition is optimal. It is also reported that a denser matrix is formed when CSA is added to UHPC [16]. The use of calcium aluminate cement (CAC) as a cementitious binder in UHPC was intensively investigated by Lee et al. [17], who reported that CSA-based UHPC is capable of withstanding heat exposure and is free from spalling, whereas typical UHPC is expected to completely lose its form at an exposure temperature ~400 °C.

The effect of blast furnace slag (BFS) incorporation in UHPC can vary depending on the curing age. The incorporation of BFS in UHPC is generally known to decrease the compressive strength at early age (1–3 days) by 18–39% [18], while the strength can be higher in the BFS-containing UHPC in comparison with the plain one after 28 days [19,20]. The effect of BFS on the properties of UHPC can also vary depending on its replacement ratios. A study by Abdulkareem et al. [21] suggests that incorporation of BFS accelerates the hydration and refines the pore structure, while a high content of BFS may reduce the compressive strength by decreasing the amount of C-S-H in UHPC. The use of metakaolin (MK) in UHPC has been found to improve the early-age strength while decreasing the later-age strength [22]. The loss in the strength when MK is used to replace PC can be 11.8% relative to the strength of the control sample after 28 days of curing [22]. On the other hand, the use of very fine MK (so-called nano MK) is reported to increase the strength by 7.9% at the sample age of 28 days at a dosage as low as 1% [23]. Materials obtained from other sources can also be used to make UHPC (i.e., demolition waste [24], mine tailings [11], cement kiln dust, rice husk ash [25]) and achieve performance comparable to the ordinary system.

Despite an increasing number of studies having investigated the mechanical properties of UHPC incorporating cementitious binders other than PC, their microstructural information, which dictates the evolution of their properties, and performance is rarely available in the literature. Therefore, this study conducts thermodynamic simulations to predict the phase assemblages of UHPC in which PC is replaced with other cementitious binders, including CAC, CSA, MK or BFS.

## 2. Materials and Methods

The mix proportion of UHPC used in this work is shown in Table 1. The water-to-cement and water-to-binder ratios of the modeled UHPC were 0.2 and 0.16, respectively. The cement denotes PC, which was gradually replaced by either CAC, CSA, MK or BFS in the simulation. The compositions (Table 2) and reaction degrees of the binders reported in previous studies were used (PC [26], CAC [27], CSA [26], MK [28] and BFS [29]). The phase assemblages of UHPC mixtures incorporating PC, CAC, CSA, MK or BFS were predicted using GEM-Selektor v3.7 [30,31] and Cemdata18 [32].

## 3. Results

### 3.1. CAC-Containing UHPC

**Figure 1:**
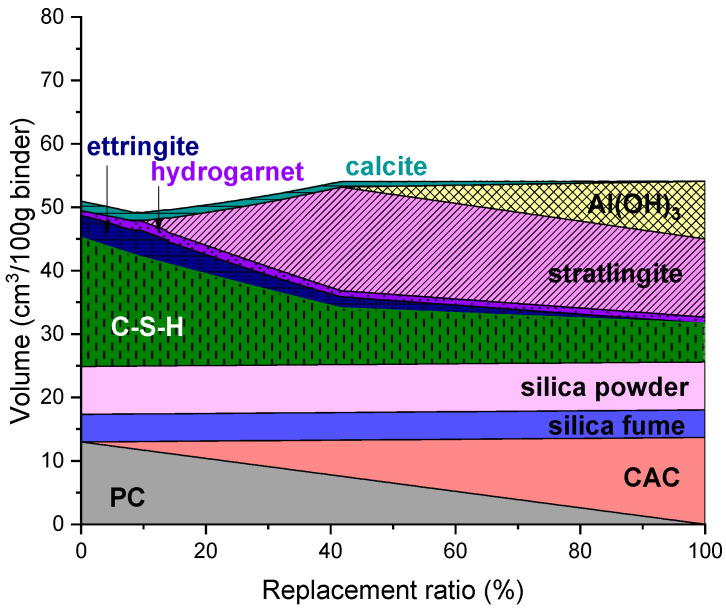
Predicted phase assemblage of UHPC containing CAC.

The predicted phase assemblage of UHPC containing CAC is shown in Figure 1. The phases which were predicted as stable in neat UHPC are C-S-H, ettringite, Fe-hydrogarnet and calcite. It is noted that portlandite was found to be unstable in all mixtures. Replacing PC with CAC in UHPC of up to 10% by mass resulted in the formation of more ettringite and Fe-hydrogarnet but reduced the amount of C-S-H and the overall volume of solid phases. The modeling result suggested that the overall volume of solid phases would increase when the replacement ratio is above 10%, due to the formation of strätlingite. The formation of strätlingite and the increase in the solid volume continued up to a 42% replacement ratio, where formation of Al(OH)_3_ begins; the increase in the solid volume continues, but at a much lower rate, due to the gradual formation of Al(OH)_3_ and the reduced strätlingite formation.

### 3.2. CSA-Containing UHPC

**Figure 2:**
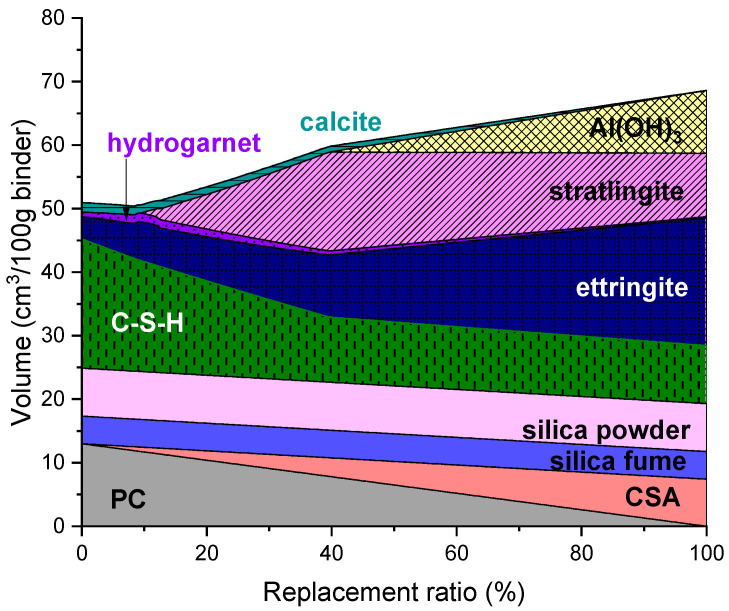
Predicted phase assemblage of UHPC containing CSA.

The predicted phase assemblage of UHPC containing CSA is shown in Figure 2. The phases which are predictedas stable in the CSA-containing UHPC were similar in the case of the CAC-containing mixture, while their volumes varied. In general, the volume of ettringite was higher, and accordingly the volume of C-S-H was predicted to be lower in the CSA-containing UHPC. The formation of strätlingite and Al(OH)_3_ in the CSA-containing UHPC was observed at similar replacement ratios that resulted in formation of those phases in the CAC-containing UHPC. The overall solid volume sharply increased at a 10% CSA replacement ratio, which coincides with the formation of strätlingite, similar to the CAC-containing UHPC. The increase in the solid volume continued even after the formation of Al(OH)_3_ started in the CSA-containing UHPC, unlike the case of the CAC-containing UHPC, which can be associated with the continued formation of ettringite.

### 3.3. MK-Containing UHPC

**Figure 3:**
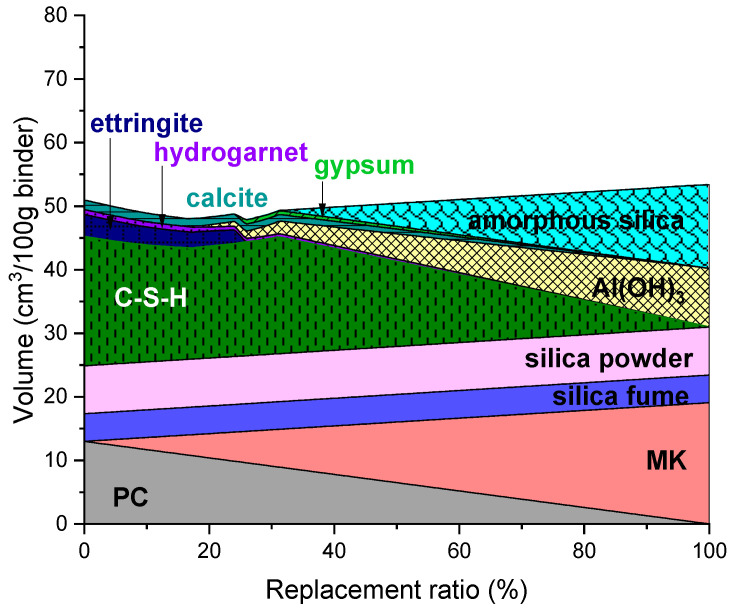
Predicted phase assemblage of UHPC containing MK.

The predicted phase assemblage of UHPC containing MK is shown in Figure 3. Replacing PC with MK in UHPC initially decreased the amount of C-S-H that forms in the mixture. Unlike those containing CSA or CAC, ettringite was found stable up to 25% replacement. In addition, the amount of Fe-hydrogarnet gradually decreased as less PC was used. The formation of Al(OH)_3_ and amorphous silica was initiated at 17% and 31% replacement ratios, respectively. The formation of amorphous silica in MK-containing UHPC indicates that the proportion of SiO_2_ in the mixture can be excessive; thus, replacing MK beyond this ratio (31%) may not be beneficial. The modeling result suggested that gypsum may precipitate at the replacement ratio of 25–90%.

The predicted volume change in the MK-containing UHPC was relatively lower than what was expected for the CSA- and CAC-containing UHPC. The overall solid volume was predicted to gradually decrease up to ~25% replacement and start increasing beyond this replacement level.

### 3.4. BFS-Containing UHPC

**Figure 4:**
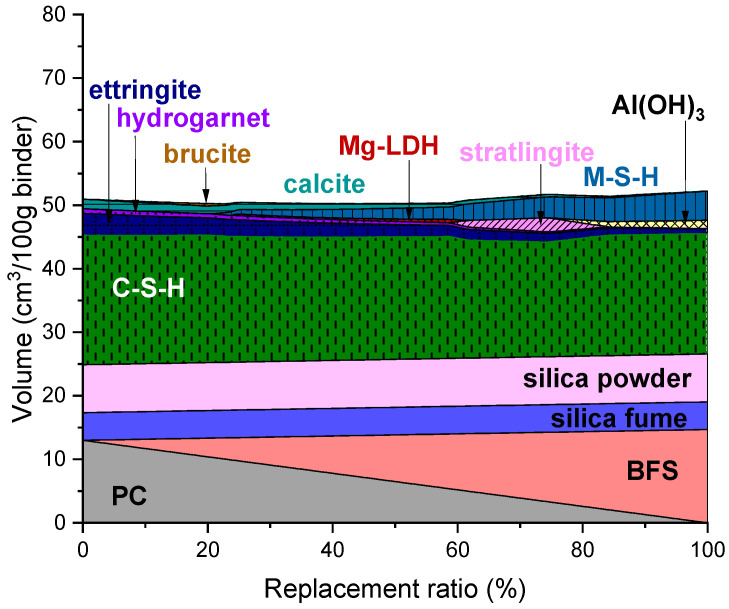
Predicted phase assemblage of UHPC containing BFS.

The predicted phase assemblage of UHPC containing BFS is shown in Figure 4. It is noticed that replacing PC with BFS brought the fewest changes in the phase assemblage in comparison with the other mixture combinations. Replacing PC with BFS in UHPC resulted in a marginal decrease in the volume of C-S-H and a more notable decrease in ettringite.

There were a number of Mg-bearing phases observed in the simulation. Brucite and Mg-LDH were predicted to form as transient phases, which are stable at 1–25% and 23–62% replacement ratios, respectively. These Mg-bearing phases were expected to convert into M-S-H at higher replacement ratios. Strätlingite was found stable at 59–84% replacement and expected to destabilize to Al(OH)_3_ and M-S-H at higher replacement levels.

The predicted volume change throughout all replacement ratios was the lowest of the prediction results presented in this work. This may be associated with the phase assemblage in the BFS-containing UHPC, which remains almost unchanged upon replacement of PC.

## 4. Discussion

**Figure 5:**
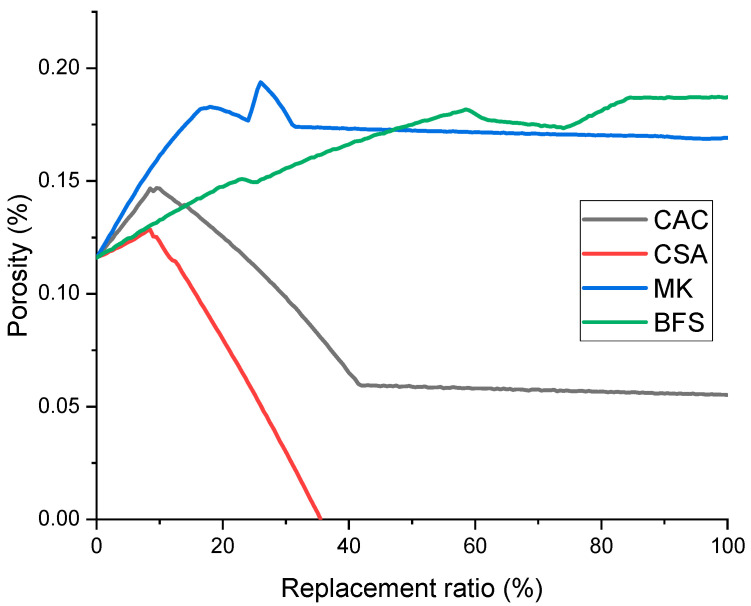
Predicted porosity as a function of replacement ratios.

The thermodynamic modeling results imply that the porosity evolution would vary dramatically according to the cementitious binders that were used to replace PC and their dosages (Figure 5). The prediction results suggest that when the dosage of CAC and CSA is greater than 24 and 12%, respectively, the mixtures are expected to have lower porosity compared to the UHPC solely consisting of PC. Replacing PC with CSA is expected to have the lowest porosity among all simulated mixtures and can induce expansion problems when the replacement ratio exceeds 35%, according to the simulation.

**Figure 6:**
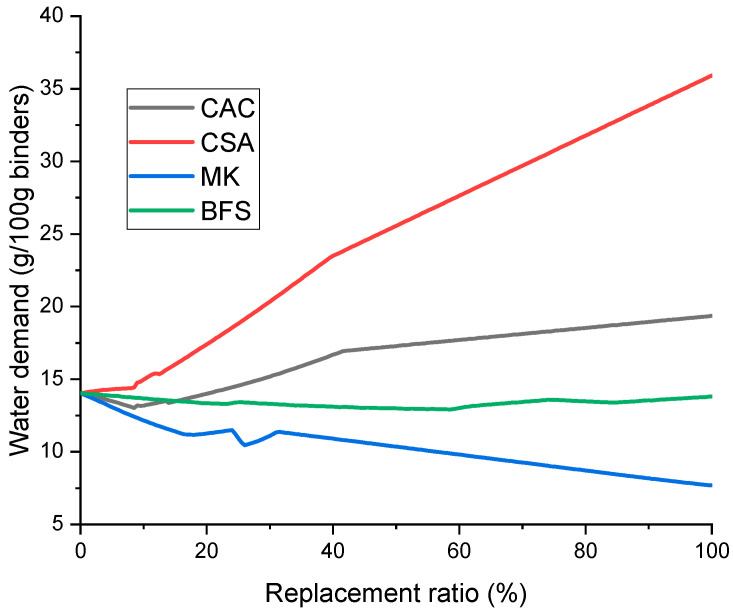
Predicted water demand of UHPC mixtures as a function of replacement ratios.

The water demand of the mixtures is simulated in Figure 6 as a function of replacement ratios. Note that the predicted water demand is the amount of water needed for chemical reactions during hydration as predicted by thermodynamic calculations; thus, it is not related to the amount of water needed to ensure homogeneous mixing. The obtained results showed some correlations with the predicted porosity, suggesting that higher water demand leads to generating lower porosity in the matrix. It is advisable that more water is added to the mixtures incorporating CAC or CSA in replacement of PC, because these binders are predicted to consume more water; otherwise, they are known to cause expansion at later ages due to the hydration of anhydrous clinkers at hardened states [26,33,34].

It is important that the results reported in this work should be cross-checked with the test results before being implemented in practice. The experimental tests for UHPC consisting of alternative cementitious binders will therefore be conducted in forthcoming studies.

## 5. Conclusions

This study investigated the effect of using alternative cementitious binders in UHPC by adopting thermodynamic calculations. Replacing PC in UHPC with CAC, CSA, MK or BFS is expected to result in significant variations in the phase assemblages. The main outcomes of this study can be summarized as follows.

(1) Strätlingite is predicted as a predominant phase in both CAC- and CSA-containing UHPC. Ettringite would increasingly form as PC is replaced with CSA.
(2) The volume of C-S-H is expected to notably decrease when replacing PC with MK, while this was not the case with BFS-containing UHPC. C-S-H remained as a predominant phase in BFS-containing UHPC throughout all replacement ratios.
(3) The predicted water demand per binder mass suggests that more water is needed for chemical reactions when using CSA and CAC, while a similar or lower amount of water is needed when using BFS and MK as a replacement of PC.
(4) It is expected that use of CSA or CAC would lead to decreasing the porosity in UHPC, while using BFS or MK may increase the porosity in comparison with that solely consisting of PC.

## Figures and Tables

**Table 1:** Mix proportion of UHPC.

**Materials**	Cement	Silica Fume	Silica Powder	Water
**Mass Ratio**	1.00	0.25	0.25	0.20

**Table 2:** Oxide compositions (mass-%) of raw materials obtained from other studies.

Oxides	PC [26]	CAC [27]	CSA [26]	MK [28]	BFS [29]
CaO	60.7	36.6	41.8		41.6
SiO_2_	20.6	4.1	8.5	52.0	36.6
Al_2_O_3_	5.0	40.3	30.4	43.8	12.2
Fe_2_O_3_	3.4	16.3	2.1	0.3	0.9
SO_3_	2.4	0.3	12.0	0.1	0.6
Na_2_O	0.2		0.1	0.3	0.2
K_2_O	1.0		0.3	0.1	0.3
MgO		0.1	2.2		7.1
SrO	0.1		0.1		
TiO_2_		1.8	1.5	1.5	
P_2_O_5_		0.2		0.2	
MnO					0.1

## Data Availability

The data presented in this study are available on request.
